# Identification and Analysis of Differentially Expressed Genes Associated with Ferroptosis and HIV in PASMCs Based on Bioinformatics

**DOI:** 10.2174/011570162X304876240821062047

**Published:** 2024-08-26

**Authors:** Tong Lu, Linna Guo, Yong Ma, Lijie Yao, Li Li, Wenshan Bian, Miao Xiu, Yang Jiang, Yongtao Li, Haifeng Jin

**Affiliations:** 1 College of Medical Technology, Qiqihar Medical University, Qiqihar, China;; 2 Department of Anatomy, Qiqihar Medical University, Qiqihar, China

**Keywords:** HIV, pulmonary arterial hypertension, ferroptosis, pulmonary arterial smooth muscle cell, IL6, PTGS2

## Abstract

**Background:**

HIV-associated pulmonary arterial hypertension (HIV-PAH), a rare and fatal condition within the pulmonary arterial hypertension spectrum, is linked to HIV infection. While ferroptosis, an iron-dependent cell death form, is implicated in various lung diseases, its role in HIV-PAH development remains unclear.

**Methods:**

Leveraging Gene Expression Omnibus data, we identified differentially expressed genes (DEGs) in pulmonary arterial smooth muscle cells, including HIV-related DEGs (HIV-DEGs) and ferroptosis-related HIV-DEGs (FR-HIV-DEGs). PPI network analysis of FR-HIV-DEGs using CytoHubba in Cytoscape identified hub genes. We conducted functional and pathway enrichment analyses for FR-HIV-DEGs, HIV-DEGs, and hub genes. Diagnostic value assessment of hub genes utilized ROC curve analysis. Key genes were further screened, and external validation was performed. Additionally, we predicted a potential ceRNA regulatory network for key genes.

**Results:**

1372 DEGs were found, of which 228 were HIV-DEGs, and 20 were FR-HIV-DEGs. TP53, IL6, PTGS2, IL1B (downregulated), and PPARG (upregulated) were the five hub genes that were screened. TP53, IL6, and IL1B act as ferroptosis drivers, PTGS2 as a ferroptosis marker, and PPARG as a ferroptosis inhibitor. Enrichment analysis indicated biological processes enriched in “response to oxidative stress” and pathways enriched in “human cytomegalovirus infection.” Key genes IL6 and PTGS2 exhibited strong predictive value *via* ROC curve analysis and external validation. The predicted ceRNA regulatory network identified miRNAs (has-mir-335-5p, has-mir-124-3p) targeting key genes and lncRNAs (XIST, NEAT1) targeting these miRNAs.

**Conclusion:**

This study advances our understanding of potential mechanisms in HIV-PAH pathogenesis, emphasizing the involvement of ferroptosis. The findings offer valuable insights for future research in HIV-PAH.

## 1 INTRODUCTION

According to the 2022 ESC/ERS guidelines for the diagnosis and treatment of pulmonary hypertension (PH), PH is defined as a mean pulmonary arterial pressure ≥ 20 mmHg and is classified into 5 groups, with pulmonary arterial hypertension (PAH) belonging to group 1 [1]. PAH is a severe and progressive cardiopulmonary disease characterized by elevated pulmonary arterial pressure and structural remodeling of pulmonary vessels, leading to right ventricular remodeling, failure, and mortality [2]. Human immunodeficiency virus (HIV)-associated PAH (HIV-PAH) is a subcategory of PAH caused by HIV infection, which is a rare but fatal disease [3]. The pathogenesis of HIV-PAH is complex and not fully understood, involving viral proteins, chronic inflammation, immune dysfunction, and oxidative stress [4]. Although the vascular pathology observed in HIV-PAH is almost identical to that observed in another subcategory of PAH, idiopathic PAH (IPAH), patients with HIV are several thousand times more likely to develop HIV-PAH compared to the incidence of IPAH [5, 6]. Moreover, HIV-PAH exhibits higher mortality rates than other HIV-related complications and serves as an independent predictor of death [7]. Pulmonary artery smooth muscle cells (PASMCs) are a key component of the arterial vascular wall, and the excessive proliferation of PASMCs is a major factor leading to pulmonary arterial remodeling [8]. While it has been reported that the HIV protein transactivator of transcription promotes PASMCs proliferation through the Notch3/VEGF-A axis [9], research on the relationship between HIV and PASMCs proliferation remains limited.

Ferroptosis is a recently discovered iron-dependent programmed cell death pathway distinct from necrosis, apoptosis, pyroptosis, and autophagy [10]. First described by Stockwell in 2012, ferroptosis is characterized by intracellular iron accumulation and excessive lipid peroxidation [11]. Numerous studies have implicated ferroptosis in the pathogenesis of various diseases, including cancer, cardiovascular diseases, and neurological disorders [10, 12, 13]. Abnormal regulation of ferroptosis is also closely associated with the development of lung diseases, such as lung cancer, chronic obstructive pulmonary disease, pulmonary fibrosis, and asthma [14]. In PAH, clinical evidence suggests that intravenous iron supplementation can effectively improve the quality of life and exercise endurance of patients with PAH, indicating that iron homeostasis imbalance may be prevalent in PAH patients [15, 16]. However, the specific role of ferroptosis in the pathological development of PAH, particularly in HIV-associated PASMCs proliferation in HIV-PAH, remains unclear and warrants further investigation.

With the advancement of gene chips and high-throughput sequencing technologies, bioinformatics methods have become more efficient and accurate in identifying key genes involved in disease development for diagnosis and treatment. In this study, we first identified the differentially expressed genes (DEGs) in PASMCs between IPAH and normal control groups using the GSE144274 dataset obtained from the Gene Expression Omnibus (GEO) database. Subsequently, we identified DEGs related to both ferroptosis and HIV (FR-HIV-DEGs) by integrating HIV-related genes from the GeneCards and DisGeNET databases and ferroptosis-related genes from the FerrDb V2 database. Functional and pathway enrichment analyses, as well as protein-protein interaction (PPI) network analysis, were performed on FR-HIV-DEGs, followed by hub gene selection using the CytoHubba plugin in Cytoscape. Additionally, we selected key genes through receiver operating characteristic (ROC) curve analysis of hub genes and validated them externally using additional datasets. Finally, miRNA and lncRNA prediction was conducted for the key genes. Our study aims to provide a better understanding of the potential mechanisms underlying HIV-PAH pathology and the involvement of ferroptosis in this process, offering new insights for HIV-PAH research.

## 2 MATERIALS AND METHODS

### 2.1 Data Collection

All datasets used in this study were obtained from the GEO database (https://www.ncbi.nlm.nih.gov/gds). Since there were no lung tissue or PASMCs samples available specifically from HIV-PAH patients, we utilized the GSE144274 dataset, which includes PASMCs samples from normal control subjects and IPAH patients, due to the similarities in vascular pathology changes between HIV-PAH and IPAH. Additionally, we retrieved HIV-associated genes from the GeneCards database v5.14 (relevance score > 2, https://www.genecards.org/) and the DisGeNET database v7.0 (https://www.disgenet.org/) using the keyword “HIV”. After removing duplicate genes, a total of 2146 HIV-associated genes were integrated. For ferroptosis-related genes, including driver factors, inhibitory factors, and markers, we downloaded 431 genes from the FerrDb V2 database (http://www.zhounan.org/ferrdb/), which is the first database specifically designed for ferroptosis regulatory factors and ferroptosis-disease associations.

### 2.2 Identification of DEGs, HIV-DEGs, and FR-HIV-DEGs

The TPM data from the GSE144274 dataset were downloaded, and DEGs between PASMCs from IPAH patients and normal control groups were identified using the limma package v3.52.4 in R software v4.2.1. The screening criteria were set as *P* value < 0.01 and |logFC| > 1. By intersecting HIV-related genes and DEGs, we obtained HIV-related DEGs (HIV-DEGs). Similarly, by intersecting ferroptosis-related genes, HIV-related genes, and DEGs, we obtained FR-HIV-DEGs. The Venn diagram online tool (http://bioinformatics.psb.ugent.be/webtools/Venn/) was used to construct Venn diagrams to visualize the results.

### 2.3 PPI Network Analysis

To predict protein-protein interactions, we utilized the STRING database v11.5 (https://string-db.org/) to retrieve protein interactions in the PPI network analysis. A threshold of interaction score > 0.4 was set, and Cytoscape v3.9.1 was used for network visualization. Nodes represent genes, and edges represent interactions between genes. The CytoHubba plugin in Cytoscape was employed to select hub genes based on the MCC algorithm.

### 2.4 Functional and Pathway Enrichment Analysis

For Gene Ontology (GO) functional enrichment analysis and Kyoto Encyclopedia of Genes and Genomes (KEGG) pathway enrichment analysis, we employed the R package clusterProfiler v3.14.3. A significance level of *P* < 0.05 and False Discovery Rate (FDR) < 0.25 was considered statistically significant. Additionally, to accurately identify pathways associated with target genes, we used the Metascape tool (http://metascape.org), a widely used enrichment analysis tool, for KEGG pathway enrichment analysis. These analysis tools utilize different algorithms, providing mutual validation.

### 2.5 ROC Curve Analysis and External Validation

Since there were no specific datasets in the GEO database for HIV-PAH patient lung tissue or PASMCs, we utilized the GSE15197 dataset containing IPAH patient and normal control lung tissue samples, as well as the GSE68563 dataset containing HIV patient and normal control peripheral blood mononuclear cell (PBMC) samples, to perform ROC curve analysis of the hub genes. The R package pROC v1.18.0 was used for this analysis. Hub genes with an ROC curve area under the curve (AUC) ≥ 0.7 in both datasets were considered key genes. Furthermore, the expression of the key genes was validated using the GSE2559 dataset containing IPAH patient and normal control PASMCs samples and the GSE140713 dataset containing HIV patient and normal control PBMC samples. Statistical differences in gene expression were analyzed using an independent samples *t*-test, with *P* < 0.05 considered statistically significant.

### 2.6 Prediction of miRNA and lncRNA

To predict miRNA targeting of key genes, we utilized the MiRTarBase database (https://mirtarbase.cuhk.edu.cn/) and Tarbase database v.8 (http://microrna.gr/tarbase/). The predicted results from these databases were intersected to ensure accuracy. For the prediction of upstream molecule lncRNAs of miRNAs, we used StarBase v2.0 (https://starbase.sysu.edu.cn/index.php/) with a threshold of very high stringency (> 5) for filtering.

## 3 RESULTS

### 3.1 Identification of DEGs, HIV-DEGs, and FR-HIV-DEGs

**Fig. (1):**
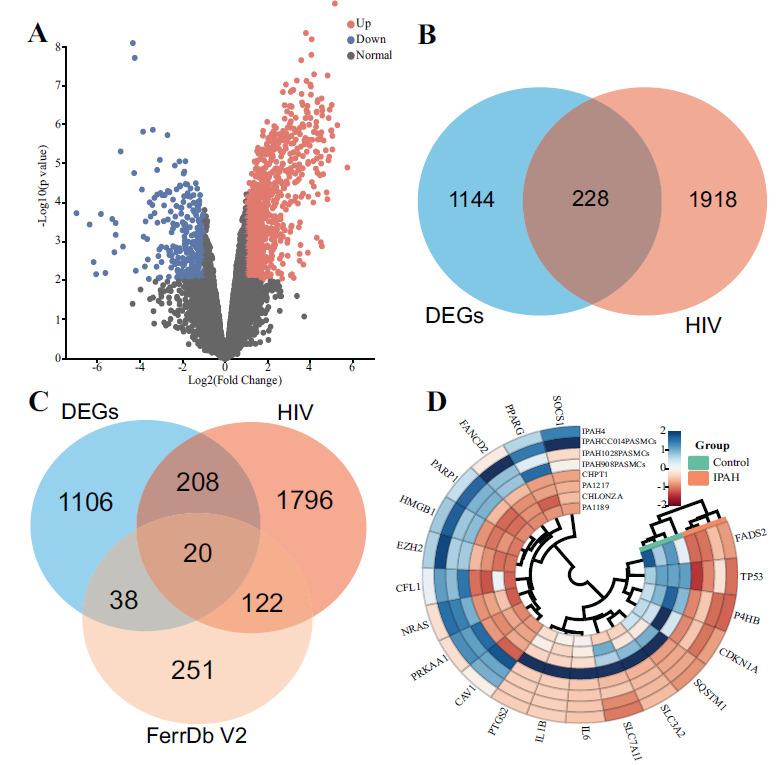
Identification of DEGs, HIV-DEGs, and FR-HIV-DEGs. (**A**) Volcano plot showing DEGs in PASMCs from IPAH patients and normal controls. Red represents upregulated genes, blue represents downregulated genes. (**B**) Venn diagram depicting HIV-DEGs. (**C**) Venn diagram depicting FR-HIV-DEGs. (**D**) Heatmap displaying FR-HIV-DEGs.

From the analysis of the GSE144274 dataset, which includes IPAH and normal control-derived PASMCs, a total of 1292 DEGs were identified, with 1008 upregulated genes and 284 downregulated genes (Fig. **1A**). The intersection of HIV-related genes and DEGs yielded 228 HIV-DEGs (Fig. **1B**). The intersection of ferroptosis-related genes, HIV-related genes, and DEGs resulted in 20 FR-HIV-DEGs (Fig. **1C**). The heatmap of the FR-HIV-DEGs displayed variations in relative gene expression in PASMCs (Fig. **1D**). The details of DEGs, HIV-DEGs, and FR-HIV-DEGs are provided in the (Supplementary material **1**).

### 3.2 Enrichment analysis of FR-HIV-DEGs and HIV-DEGs

**Fig. (2):**
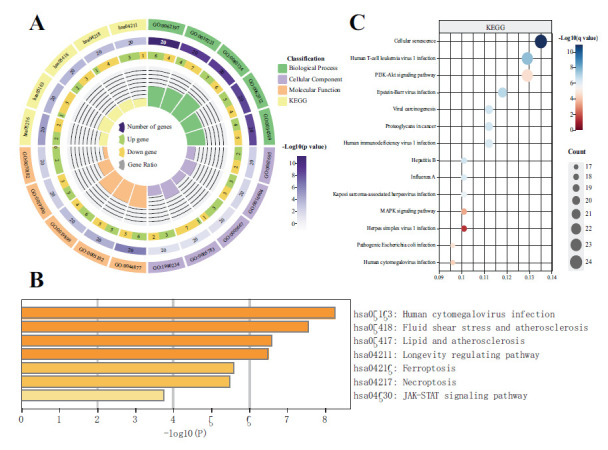
Enrichment analysis of FR-HIV-DEGs and HIV-DEGs. (**A**) GO and KEGG enrichment analysis results of FR-HIV-DEGs using R software. (**B**) KEGG enrichment analysis results of FR-HIV-DEGs using Metascape enrichment analysis tool. (**C**) KEGG enrichment analysis results of HIV-DEGs using R software. *P* < 0.05 was considered significant.

GO and KEGG enrichment analyses were performed to investigate the potential biological functions and related pathways of FR-HIV-DEGs using R software (Fig. **2A**). The GO analysis revealed significant enrichment in biological processes, such as “cellular response to chemical stress” and “response to oxidative stress”. KEGG analysis showed significant enrichment in pathways such as “human cytomegalovirus infection” and “microRNAs in cancer.” Additionally, using the Metascape online tool for KEGG pathway enrichment analysis, significant enrichment in pathways, such as “human cytomegalovirus infection”, “fluid shear stress and atherosclerosis”, and “lipid and atherosclerosis” was observed (Fig. **2B**). Moreover, the pathways enriched by HIV-DEGs were analyzed, and “human cytomegalovirus infection” was found to be significantly enriched (Fig. **2C**).

### 3.3 PPI Network Analysis of FR-HIV-DEGs and Identification of Hub Genes

**Fig. (3):**
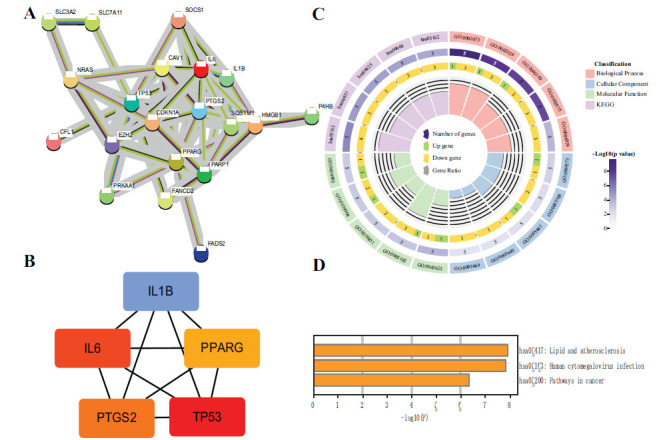
Construction of PPI network, identification of hub genes, and enrichment analysis of hub genes. (**A**) PPI network of FR-HIV-DEGs comprising 20 nodes and 59 edges. (**B**) Network illustrating the top 5 hub genes. (**C**) GO and KEGG enrichment analysis results of hub genes using R software. (**D**) KEGG enrichment analysis results of hub genes using Metascape enrichment analysis tool.

The 20 FR-HIV-DEGs were used to construct a PPI network using the STRING database, resulting in a network of 20 nodes and 59 edges (Fig. **3A**). The top 5 hub genes based on the MCC score were identified using the CytoHubba plugin, including 4 downregulated genes (TP53, IL6, PTGS2, IL1B) and 1 upregulated gene (PPARG) (Fig. **3B**). Enrichment analysis of the hub genes revealed their involvement in biological processes such as “regulation of acute inflammatory response” and “response to oxidative stress,” according to GO analysis. KEGG analysis showed significant enrichment in pathways such as “human cytomegalovirus infection” and “IL-17 signaling pathway” (Fig. **3C**). Furthermore, Metascape pathway enrichment analysis identified significant enrichment in the “lipid and atherosclerosis” and “human cytomegalovirus infection” pathways (Fig. **3D**).

Based on the enrichment analysis results of FR-HIV-DEGs, HIV-DEGs, and hub genes, it was observed that the biological process “response to oxidative stress” and the pathway “human cytomegalovirus infection” were enriched in all three sets of results.

### 3.4 ROC Curve Analysis and Identification of Key Genes and External Validation

**Fig. (4):**
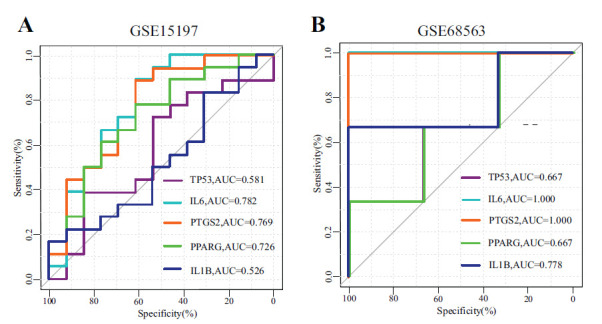
ROC curve analysis of hub genes. (**A**) ROC curve of hub genes in the GSE15197 dataset. (**B**) ROC curve of hub genes in the GSE68563 dataset.

**Fig. (5):**
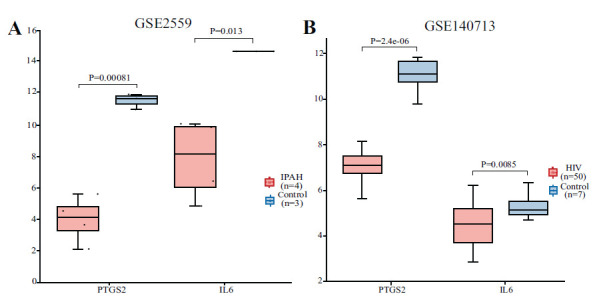
External validation of key genes. (**A**) Expression levels of IL6 and PTGS2 in the GSE2559 dataset. (**B**) Expression levels of IL6 and PTGS2 in the GSE140713 dataset.

ROC curve analysis was performed using datasets GSE15197 and GSE68563 to evaluate the diagnostic value of the hub genes (Fig. **4**). The results showed that IL6 and PTGS2 had the highest AUC in both datasets, with values of 0.782 and 0.769 in GSE15197, and both were 1 in GSE68563. This indicates that IL6 and PTGS2 have high specificity and sensitivity. Therefore, IL6 and PTGS2 were defined as the key genes in this study. The key genes were further validated using datasets GSE2559 and GSE140713, demonstrating statistically significant differential expression (*P* < 0.05) (Fig. **5**).

### 3.5 LncRNA-miRNA-mRNA Interaction Network

**Fig. (6):**
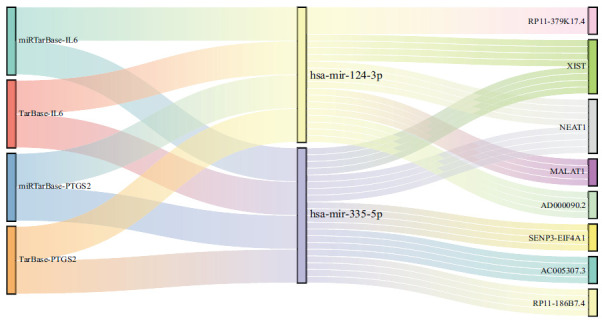
Sankey diagram illustrating the relationship between mRNAs, miRNAs, and lncRNAs.

A potential regulatory network involving competing endogenous RNA (ceRNA) for the key genes was predicted (Fig. **6**). MiRNA prediction for IL6 and PTGS2 was performed using the MiRTarBase and Tarbase databases, and overlapping miRNAs were selected. The intersection of predicted miRNAs targeting IL6 and PTGS2 resulted in two key miRNAs, has-mir-335-5p and has-mir-124-3p. Using StarBase 2.0, upstream lncRNAs targeting these two key miRNAs were predicted, resulting in two lncRNAs, XIST and NEAT1, which simultaneously target these miRNAs.

## 4 DISCUSSION

As highly active antiretroviral therapy continues to improve the health of HIV patients and reduce mortality rates, the focus of disease management has shifted from treating opportunistic infections related to immune deficiency to managing chronic cardiovascular and pulmonary complications [17, 18]. HIV-PAH is a recognized and serious complication of HIV infection, associated with significant morbidity and mortality [6]. Recognizing the significance of HIV-PAH is crucial, as it is likely to become the most common cause of PAH worldwide [6]. However, the exact pathogenesis of HIV-PAH remains unclear, necessitating further research to elucidate its underlying mechanisms, facilitate early diagnosis, and identify treatment targets to improve the prognosis of this serious complication.

In this study, we identified 228 HIV-DEGs and 20 FR-HIV-DEGs, including 5 hub genes (TP53, IL6, IL1B, PTGS2, and PPARG), to investigate the mechanisms of PASMCs proliferation and its relationship with ferroptosis in HIV-PAH. TP53, IL6, and IL1B are ferroptosis drivers, and in the dataset we analyzed (GSE144274), these genes were downregulated among the DEGs. PTGS2, a marker of ferroptosis, also showed downregulation, whereas PPARG, an inhibitor of ferroptosis, was upregulated (Additional file 1). These gene expression patterns indicate that, compared to PASMCs from normal controls, the PASMCs from IPAH patients showed a downregulation of ferroptosis drivers and markers and an upregulation of ferroptosis inhibitors. Therefore, this finding suggests that the ferroptosis process may be inhibited in the context of excessive proliferation in HIV-associated PASMCs. In addition, enrichment analysis of FR-HIV-DEGs, HIV-DEGs, and hub genes revealed significant enrichment in biological processes related to “response to oxidative stress” and pathways associated with “human cytomegalovirus infection”. There is a substantial relationship between cytomegalovirus (CMV) infection and oxidative stress. For instance, in the development of atherosclerotic lesions following CMV infection, early gene products of CMV, IE72 and IE84 induce the production of reactive oxygen species (ROS) through a cyclooxygenase-2 (COX-2)-dependent mechanism, leading to vascular endothelial cell damage [19].

Additionally, CMV activates NADPH oxidase in vascular smooth muscle cells, increasing ROS production and subsequently promoting excessive proliferation of these cells [19]. ROS are also believed to play a role in activating the CMV replication process, affecting the viral replication cycle. Studies have shown that the upregulation of ROS enhances the transcription and protein expression of CMV genes pp72 and pp65, while the use of ROS scavengers can effectively inhibit CMV replication [20]. Therefore, antioxidants and related strategies targeting ROS inhibition may be potentially effective antiviral treatments for CMV infection. In addition, oxidative stress is closely linked to both ferroptosis and the pathological development of PAH. The enrichment of biological processes related to “response to oxidative stress” further indicates that ferroptosis is involved in the pathological changes of HIV-related PASMCs. Furthermore, it is worth noting that CMV poses a significant health risk to immunocompromised individuals such as HIV-infected patients and developing fetuses [21]. It has been reported that 50% of AIDS patients who underwent autopsy were found to have CMV infection, with approximately 45% of them involving the lungs, indicating that HIV patients are susceptible to CMV infection, which may further promote lung injury [22]. Moreover, several case reports have demonstrated that CMV infection is associated with severe respiratory distress and persistent pulmonary hypertension [23-25]. Although a direct relationship between the pathological development of HIV-PAH and CMV infection has not been definitively established, the pathway analysis results and existing evidence suggest that HIV and CMV may share similar mechanisms in the development of PAH, with CMV infection potentially playing a significant role in the pathogenesis of HIV-PAH. However, further in-depth research is needed to confirm this.

Next, we conducted ROC curve analysis and external validation to assess the hub genes further. We identified IL6 and PTGS2 as the key genes with good predictive value. IL-6, an important inflammatory cytokine, is highly expressed in the plasma and lung tissue of PAH patients and promotes excessive proliferation of PASMCs, which is positively correlated with disease severity [26]. In HIV-PAH, IL-6 is considered one of the main cytokines responsible for PAH development [27]. The plasma level of IL-6 has prognostic value in PAH-HIV, independent of functional or hemodynamic parameters [17]. In this study, we found that the expression level of the IL6 gene, a driver of ferroptosis, was decreased in the FR-HIV-DEGs. During the pathological progression of HIV-PAH, high plasma levels of IL-6 promote the proliferation of PASMCs, while the ferroptosis process in PASMCs is inhibited. These factors may contribute to the excessive proliferation of PASMCs together. PTGS2, which encodes COX-2, plays a crucial role in mediating downstream prostaglandin production, including PGE2 and PGI2, which have significant anti-proliferative effects in PAH [28]. Studies have shown that COX-2-deficient mice develop severe pulmonary hypertension under hypoxic conditions, accompanied by vascular remodeling and right ventricular hypertrophy [29]. COX-2 is considered a valuable biomarker for PAH [30]. Previous research on PTGS2 and COX-2 in the context of HIV has primarily focused on neurologic disorders associated with HIV infection, where increased expression levels of PTGS2 mRNA and COX-2 protein have been observed [31, 32]. However, there are no reports specifically addressing the role of PTGS2 in HIV-related pulmonary diseases. In this study, PTGS2, as a marker of ferroptosis, was found to be downregulated in FR-HIV-DEGs, suggesting that PTGS2 may play an important role in the anti-proliferation of PASMCs during HIV-PAH pathogenesis.

The ceRNA network has been recognized as a significant regulatory network in gene expression and disease pathogenesis. Many miRNAs and lncRNAs have been found to be associated with HIV infection and replication, and they affect host cell gene expression and immune response [33, 34]. Therefore, we predicted the potential miRNAs and upstream lncRNAs that may regulate the mRNA expression of IL6 and PTGS2. Two miRNAs, mir-124-3p and mir-335-5p, as well as two lncRNAs, XIST and NEAT1, were identified. Mir-124-3p and mir-335-5p have been implicated in PAH pathology and ferroptosis, but their roles in HIV infection and disease progression remain unclear [35-38]. XIST and NEAT1, on the other hand, have been associated with PAH pathology, ferroptosis, as well as HIV infection and disease progression [39-43]. While we predicted a ceRNA network comprising lncRNA-miRNA-mRNA interactions, further experimental validation is necessary to understand the underlying mechanisms and confirm their roles in the development of HIV-PAH pathology.

## CONCLUSION

In summary, this study utilized bioinformatics analysis to predict, for the first time, the DEGs in PASMCs related to HIV and ferroptosis. Key genes were identified, shedding light on their potential roles in PASMCs proliferation during the progression of HIV-PAH pathology. These findings contribute to a better understanding of HIV-PAH pathology and provide a basis for future research. However, it is important to acknowledge the limitations of our study. Selecting PASMCs datasets from HIV-infected patients would best represent the pathological changes in PASMCs following HIV infection. However, there are currently no available samples or datasets from HIV-PAH patients' PASMCs in the public gene database. To explore the relationship between HIV-related genes and PASMCs proliferation, we selected the PASMCs dataset GSE144274, which includes samples from patients with IPAH, a subgroup of PAH. This choice was based on the observed similarities in vascular pathology between HIV-PAH and IPAH. Additionally, we identified and integrated HIV-related genes from the GeneCards and DisGeNET databases, intersecting these with DEGs from the GSE144274 dataset for further analysis. This approach provides valuable insights into the potential role of HIV-related genes in PASMCs proliferation. However, we must acknowledge the limitations of these methods. While IPAH and HIV-PAH share pathological similarities, they are not entirely identical diseases. Thus, these methods may not fully reflect all the pathological changes present in HIV-PAH. Humanized mice, which contain CD4+ T cells susceptible to HIV-1 infection, have been widely used to study HIV pathogenesis, viral latency, transmission pathways, host immune responses, as well as prevention and antiviral therapies [44]. According to the research by Rodriguez-Irizarry *et al.* [45], the HIV/SU5416 model established in HIV-infected humanized mice treated with the synthetic inhibitor of vascular endothelial growth factor receptor-2 (SU5416) may be one of the best models for studying pulmonary hypertension and pulmonary vascular diseases. This model exhibits significant advantages in terms of survival rates and pulmonary hemodynamic characteristics. In future studies, we plan to develop the HIV/SU5416 humanized mouse model to validate our preliminary findings further and to explore the mechanisms of pulmonary vascular disease in HIV-PAH.

## Data Availability

All data generated or analysed during this study are included in this published article.

## LIST OF ABBREVIATIONS

FDR: False Discovery Rate
PAH: Pulmonary Arterial Hypertension
PH: Pulmonary Hypertension
IPAH: Idiopathic PAH
GEO: Gene Expression Omnibus
GO: Gene Ontology
ROC: Receiver Operating Characteristic
ROS: Reactive Oxygen Species
