# Supplementary material for: Identification and Analysis of Differentially Expressed Genes Associated with Ferroptosis and HIV in PASMCs Based on Bioinformatics
Source: Curr HIV Res. 2024 Aug 26;22(5):308–17. doi: 10.2174/011570162X304876240821062047 (PMC11826917; doi:10.2174/011570162X304876240821062047)
Supplement: Supplementary file 1 [file CHIVR-22-5-308_SD1.pdf]

Supplementary Material

Identification and Analysis of Differentially Expressed Genes Associated with Ferroptosis an d HIV in PASMCs Based on Bioinformatics

Tong Lu[1], Linna Guo[2], Yong Ma[2], Lijie Yao[2], Li Li[2], Wenshan Bian[2], Miao Xiu[2], Yang Jiang[2,#], Yong-tao Li[2,#] and Haifeng Jin[2,*]

[1]*College of Medical Technology, Qiqihar Medical University, Qiqihar, China;* [2]*Department of Anatomy, Qiqihar Medical University, Qiqihar, China*

Supplementary table 5: Pub Chem CID and structures of the ligand for molecular docking

**Structure**	**PubChem CID**	**Canonical SMILES**	**Chemical compound**	**Degree**	**Molecular formula**	**Molecular weight (g/mol)**	**XLogP3-AA**	**Hydrogen Bond Donor Count**	**Hydrogen Bond Acceptor Count**	**Rotatable Bond Count**
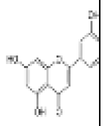	64945	C1=CC(=C(C=C1C2=CC(=O)C3=C(C=C(C=C3O2)O)O)O)O	**Luteolin**	**31**	$C_{15}H_{10}O_6$	286.24	1.4	4	6	1
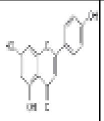	5280443	C1=CC(=CC=C1C2=CC(=O)C3=C(C=C(C=C3O2)O)O)O	Apigenin	**27**	$C_{15}H_{10}O_5$	270.24	1.7	3	5	1
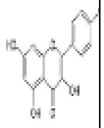	5280863	C1=CC(=CC=C1C2=C(C(=O)C3=C(C=C(C=C3O2)O)O)O)O	Kaempferol	20	$C_{15}H_{10}O_6$	286.24	1.9	4	6	1
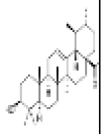	64945	CC1CCC2(CCC3(C(=CCC4C3(CCC5C4(CCC(C5(C)C)O)C)C)C2C1C)C)C(=O)O	Ursolic acid	11	$C_{30}H_{48}O_3$	456.7	7.3	2	3	1
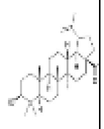	64971	CC(=C)C1CCC2(C1C3CCC4C5(CCC(C(C5CCC4(C3(CC2)C)C)(C)C)O)C)C(=O)O	Betulinic acid	3	$C_{30}H_{48}O_3$	456.7	8.2	2	3	2
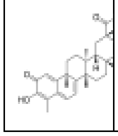	122724	CC1=C(C(=O)C=C2C1=CC=C3C2(CCC4(C3(CCC5(C4CC(CC5)(C)C(=O)O)C)C)C)C)O	Celastrol	3	$C_{29}H_{38}O_4$	450.6	5.9	2	4	1

Supplementary table 6: PDB IDs and structures of the proteins for molecular docking

Sphere object(Grid center)								
**Sr.No.**	**Target**	**Uniprot ID**	**PDB ID**	**X**	**Y**	**Z**	**Sphere radius**	**Structure**
1	Estrogen Receptor 1(ESR1)	P03372	1A52	98.230875	14.500675	84.477350	19.113949	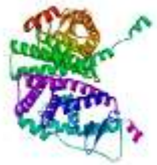
2	Prostaglandin-Endoperoxide Synthase 2 (PTGS2)	P35354	5F19	25.724542	34.626254	34.570257	48.934654	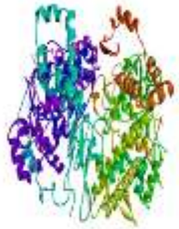
3	Cyclin Dependent Kinase 1 (CDK1)	P06493	4Y72	29.214538	-72.059269	184.798269	8.662336	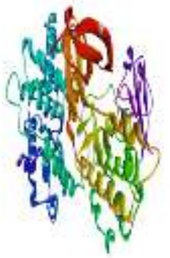
4	Cyclin B1(CCNB1)	P14635	4YC3	22.365000	20.322125	173.165562	24.807966	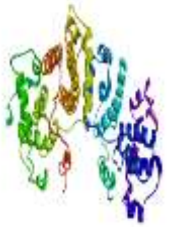
5	Cell Division Cycle 25B(CDC25B)	P30305	1CWR	11.803000	8.761250	31.417750	5.000000	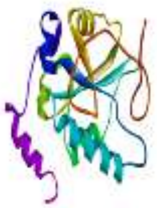
6	Matrix Metallopeptidase 9(MMP9)	P14780	1GKC	53.253727	22.512818	129.715318	23.112434	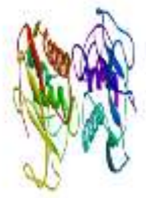

7	Cyclin Dependent Kinase 6(CDK6)	Q00534	3NUP	23.477955	35.711136	-8.069091	6.211723	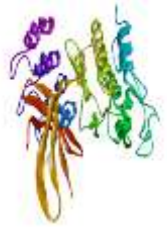
8	Cytochrome P450 Family 1 Subfamily B Member 1(CYP1B1)	Q16678	6IQ5	21.979173	-33.724245	-2.751909	40.420815	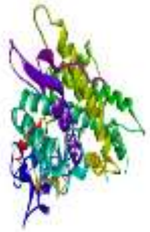
9	Protein Tyrosine Phosphatase Non-Receptor Type 1(PTPN1)	P18031	1BZC	-19.393828	54.273138	17.149897	8.470053	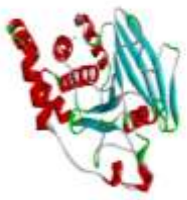
10	ATP Binding Cassette Subfamily G Member 2 (Junior Blood Group)(ABCG2)	Q9UNQ0	5NJ3	124.856411	124.753161	92.120304	24.550314	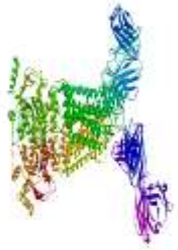

Supplementary table 7: Corresponding pharmacophore class for interaction of the active compounds with the target.

	**Pharmacophore Class**				
**Active compound with the targe**	**Hydrogen Donor**	**Hydrogen Acceptor**	**Hydrophobic**	**Aromatic**	**Negative ion**
Apigenin with the Target CDK 1	3	2	3	1	-
Kaempferol with the target CDK 1	4	3	3	-	-
Luteolin with the target CDK 1	3	2	3	-	-
Celastrol with the target in CDK6	1	-	3	-	-
Luteolin with the target CDK6	3	3	3	-	-
Apigenin with the target CDK6	2	2	2	-	-

kaempferol with the target CDK6.	3	2	3	-	-
Celastrol with the target PTPN1	1	1	-	-	-
Celastrol with the target CDC25B	1	3	-	-	1
Betulinic acid with the target CDC25B	-	2	-	-	1
